# Hypofractionated radiotherapy for newly diagnosed elderly glioblastoma patients: A systematic review and network meta-analysis

**DOI:** 10.1371/journal.pone.0257384

**Published:** 2021-11-04

**Authors:** Suely Maymone de Melo, Gustavo Nader Marta, Carolina de Oliveira Cruz Latorraca, Camila Bertini Martins, Orestis Efthimiou, Rachel Riera

**Affiliations:** 1 Neuro-Oncology–Hospital do Coração de Sao Paulo, Sao Paulo, Brazil; 2 Evidence-Based Medicine Post-graduation Program, Universidade Federal de São Paulo (Unifesp), Sao Paulo, Brazil; 3 Department of Neurosurgery Escola Paulista de Medicina, Universidade Federal de São Paulo (Unifesp), Sao Paulo, Brazil; 4 Department of Radiation Oncology—Hospital Sírio-Libanês, Sao Paulo, Brazil; 5 Hospital São Paulo, Universidade Federal de São Paulo (Unifesp), São Paulo, Brazil; 6 Department of Preventive Medicine, Escola Paulista de Medicina, Universidade Federal de São Paulo (Unifesp), Sao Paulo, Brazil; 7 Institute of Social and Preventive Medicin—Universität Bern, Bern, Switzerland; 8 Discpline of Evidence-based Medicine, Escola Paulista de Medicina, Universidade Federal de São Paulo (Unifesp), Sao Paulo, Brazil; 9 Center of Health Technology Assessment—Hospital Sírio-Libanês, Sao Paulo, Brazil; University of Zurich, SWITZERLAND

## Abstract

**Objective:**

To evaluate different hypofractionated radiotherapy (HRT) regimens for newly diagnosed elderly glioblastoma (GBM) patients.

**Methods:**

We performed a systematic review with network meta-analysis (NMA), including searches on CENTRAL, Medline, EMBASE, CINAHL, clinical trial databases and manual search. Only randomized clinical trials (RCTs) were included. Primary outcomes: overall survival (OS) and adverse events (AE). Secondary outcomes: progression-free-survival (PFS) and quality of life (QoL). We used the Cochrane Risk of Bias (RoB) table for assessing individual studies and CINeMA for evaluating the certainty of the final body of evidence.

**Results:**

Four RCTs (499 patients) were included. For OS, the estimates from NMA did not provide strong evidence of a difference between the HRTs: 40 Gray (Gy) versus 45 Gy (HR: 0.89; CI 95%: 0.42, 1.91); 34 Gy versus 45 Gy (HR: 0.85; CI 95% 0.43, 1.70); 25 Gy versus 45 Gy (HR: 0.81; CI 95% 0.32, 2.02); 34 Gy versus 40 Gy (HR: 0.95; CI 95% 0.57, 1.61); and 25 Gy versus 34 Gy (HR: 0.95; CI 95% 0.46, 1.97). We performed qualitative synthesis for AE and QoL due to data scarcity and clinical heterogeneity among studies. The four studies reported a similar QoL (assessed by different methods) between arms. One RCT reported grade ≥ 3 AE, with no evidence of a difference between arms. PFS was reported in one study (25 Gy versus 40 Gy), with no evidence of a difference between arms.

**Conclusion:**

This review found no evidence of a difference between the evaluated HRTs for efficacy and safety.

## Introduction

Glioblastoma (GBM) accounts for 14.6% of all CNS tumors in adults. It is the most common malignant CNS tumor (48.3%) and is reported as representing the majority of gliomas (57.3%). Its incidence rate increases in patients aged over 65 [1]. Elderly patients have additional co-morbidities that are associated with worse prognosis [2]. The median overall survival (OS) in this population after radiotherapy (RT) alone is around six months [3].

The concept of ‘elderly patient’ is not well established. Most authors set the cut-off at 65 years, but it can range from 60 to 70 years. These patients usually suffer from additional co-morbidities and are associated with worse prognosis. For this reason, clinical trials in patients with GBM have traditionally excluded elderly patients [2, 4–6]. Population studies have shown that elderly patients usually have less intense treatments. However, when this differential choice of treatment was accounted for in the analyses, the difference between the outcomes in the different treatments disappeared [7–10].

The initial treatment for newly diagnosed GBM patients consists of maximal safe resection. Concomitant and adjuvant Temozolomide (TMZ) have been shown to have a survival benefit when added to a standard course RT (60 Gy in 30 daily fractions, over six weeks). In the subgroup analysis of the elderly population (>60 years), they observed an increase in survival. However, due to the small number of patients evaluated (n = 170), the interaction test was underpowered to allow definitive conclusions [11, 12].

The NCIC/EORTC phase III trial assessed patients ≥ 65 years old and Eastern Cooperative Oncology Group (ECOG) performance status 0–2 and randomized them to receive concomitant HRT (40 Gy in 15 fractions) and TMZ (75 mg/m2/day), followed by adjuvant temozolomide (150–200 mg/m2, 5/28-days cycle, 12 cycles or until progression) or HRT alone (same schedule). There was an improvement in OS and PFS in the HRT plus TMZ group (9.3 months versus 7.6 months; HR = 0.67; 95%CI 0.56, 0.80; P < 0.0001 and 5.3 versus 3.9 months; HR = 0.50; 95%CI 0.41, 0.60; p < 0.001, respectively). The patients with O-6-methylguanine-DNA methyltransferase (MGMT) promoter methylation tumors had the greatest benefit in OS (13 months) [13]. This treatment is now considered the standard of care for good-prognosis elderly patients. However, due to the lack of a direct comparison between the HRT schemes, it is not yet possible to define the treatment of choice for this population. Reducing the time of treatment without compromising safety and efficacy is essential for elderly patients. Besides, fewer treatment sessions result in decreasing costs and require fewer resources [14].

This study aimed to compare the efficacy and safety of different HRT schemes for elderly patients with newly diagnosed GBM.

## Methods

### Local and design

We conducted a systematic review with network meta-analysis in the Evidence-Based Health Program, at the Universidade Federal de São Paulo, Brazil. The protocol was prospectively registered on the PROSPERO database and available from http://www.crd.york.ac.uk/PROSPERO/display_record.php?ID=CRD42018100600.

This reporting followed the PRISMA Extension Statement for Reporting of Systematic Reviews Incorporating Network Meta-analyses of Health Care Interventions (PRISMA-NMA) [15].

### Criteria for including studies

#### Type of studies

Randomized clinical trials (RCTs).

#### Type of participants

Subjects with newly diagnosed histologically confirmed GBM (WHO grade IV), aged 60 or older.

#### Type of interventions

Post-operative focal hypofractionation regimens

### Outcomes

The primary outcomes were: (a) overall survival (OS) defined as the time from diagnosis or randomization to the date of death or last follow-up, and (b) adverse events (AE) as defined by the World Health Organization (WHO) or the National Cancer Institute Common Terminology Criteria (NCI-CTC).

The secondary outcomes were: (c) progression-free survival (PFS) defined as the time from diagnosis or randomization to the date of progression (assessed by Response Assessment in Neuro-Oncology - RANO—criteria) or death, and (d) health-related quality of life (QoL), assessed by any validated tool.

### Timing of outcome assessment

We assessed all outcomes stated above at any time point. We pooled short-term (up to three months, inclusive) or long-term (more than three months) outcomes. When an author reported an outcome more than once in one interval for registering the event, we planned to consider the last measurement.

### Search strategy

We conducted highly sensitive search strategies (January 28, 2019, updated on November 09, 2020, with no new study added) for the following electronic databases: Excerpta Medica dataBASE (Embase, via Elsevier), Cochrane Central Register of Controlled Trials (CENTRAL, via Wiley), and Medical Literature Analysis and Retrieval System Online (MEDLINE, via PubMed). The full search strategies for all databases are available at **online S1 Table.** We carried out additional searches in gray literature (www.opengrey.eu) and clinical trials register databases (https://www.clinicaltrials.gov and https://www.who.int/ictrp/search/en/)). We conducted manual searches among the reference lists of included studies, review articles and proceedings of the meetings of the American Society of Radiation Oncology (ASTRO) and the European Society for Radiotherapy and Oncology (ESTRO). We did not impose restrictions on date, language or status of publication (abstract or full-text).

### Selecting studies

We selected the studies through a two-stage process. During the first stage, the titles and abstracts of the retrieved references were evaluated. In the second stage, the full texts of potentially eligible studies were scrutinized against the inclusion criteria. Both stages were carried out independently by two reviewers (SMM and GNM), and a third reviewer (RR) adjudicated in the case of divergencies We used the Rayyan platform for the selection process (https://rayyan.qcri.org/) [16].

### Collecting data

For the extraction, we used a standard data collection form for intervention reviews in RCTs only (Cochrane Library). We excluded duplicates and gathered multiple reports of the same study. Two reviewers (SMM and GN) extracted the data from the included studies. A third reviewer (RR) solved any disagreement. We obtained data on possible effect modifiers in the population (inclusion and exclusion criteria, different age ranges, baseline performance status, MGMT, surgical extent) and interventions (HRT protocols), methods (study design, number of study centers and location, duration of study, date of study, withdrawals), outcomes (primary and secondary, planned and reported) sponsorship/funding, and authors’conflicts of interest.

### Dealing with missing data

We tried to contact authors or study sponsors if missing data related to the outcomes.

### Assessing the risk of bias in included studies

Two reviewers (SMM, GNM) independently evaluated the risk of bias for each study using the Cochrane Risk of Bias (RoB) table [17]. An additional reviewer (RR) solved any disagreement.

We judged each outcome separately for the domains blinding of participants and personnel, blinding of outcome assessors and incomplete outcome data.

### Unit of analysis issues

The unit of analysis was the individual patient.

### Data synthesis and analysis

One of the underlying assumptions of NMA is transitivity [18, 19]. We assessed this assumption by comparing the distribution of the potential effect modifiers across the different pairwise comparisons, i.e. if the treatments characteristics, participants and clinical questions were deemed to be similar across treatment comparisons.

In case there was more than one study per comparison, we aimed to synthesize data using a random-effects meta-analysis model for each pairwise treatment comparison and to report the estimated heterogeneity. We performed a frequentist NMA, using the netmeta package in R [20]. We used a single parameter to model heterogeneity in the network [21], a common assumption in NMA. We used the p-score to rank the investigated hypofractionated schemes [22]. For time-to-event data, we used hazard ratios (HRs). For the toxicity analysis we aimed to use risk ratios (RRs). For the QOL analysis we aimed to use mean score difference. We reported results in terms of ‘league-tables’ and generalized forest-plots, where we show the relative effects of each treatment versus the network reference (which we chose to be 60 Gy).

We assessed the extent of statistical heterogeneity in our meta-analyses via the estimated heterogeneity variance parameter (*τ*^2^). To assess the inconsistency in the network locally, we aimed to use a back-calculation method [23]. To assess inconsistency globally, we used the design-by-treatment inconsistency model. This model accounts for design inconsistency (e.g., when two-arm and three-arm trials give different results) as well as loop inconsistency (i.e., the disagreement between direct and indirect evidence).

In case we found comparisons with ten or more studies per comparison, we aimed to produce a contour-enhanced funnel-plot to explore whether results in imprecise trials differ systematically from results in more precise trials. We aimed to use Egger’s test to test for funnel-plot asymmetry, aiming to assess the possibility of small-study effects and publication bias [24].

We used the free online tool CINeMA (Confidence in Network Meta-Analysis Software Institute of Social and Preventive Medicine, University of Bern, 2017- cinema.ispm.unibe.ch) [25] to evaluate the certainty of evidence for each pre-specified outcome (https://journals.plos.org/plosmedicine/article?id=10.1371/journal.pmed.1003082). Two authors applied the tool and divergencies were solved by a third author.

In case there were enough studies, we planned to perform subgroup analyses regarding the MGMT marker and the risk of bias.

## Results

### Search results

**Fig 1:**
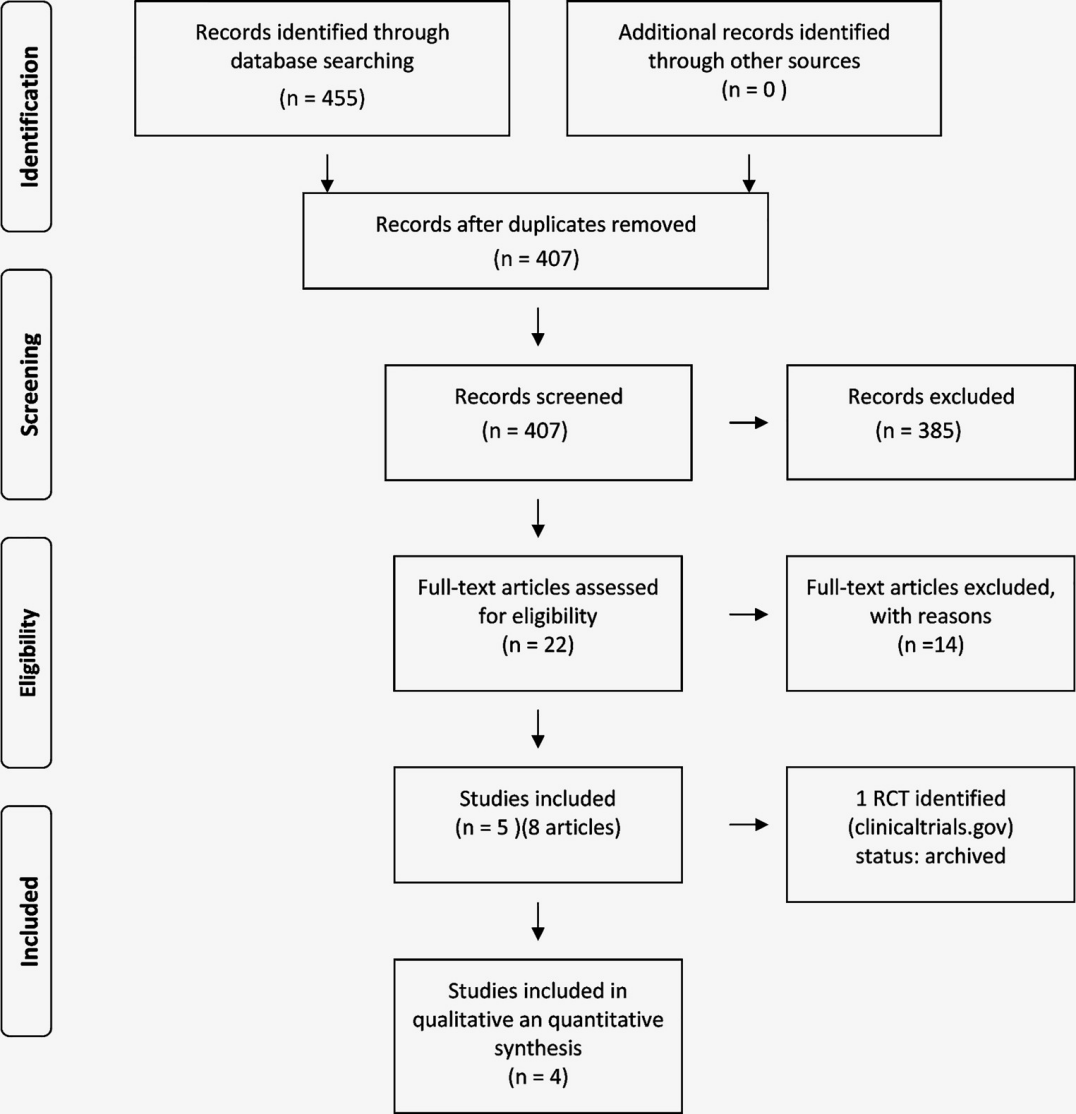
Flowchart of the studies selection process.

The initial search retrieved 455 records. After eliminating duplicates, we assessed 407 records by reading the titles and abstracts, and identified 22 records as elegible for full-text evaluation. After this stage, 14 records were excluded for various reasons (**online S2 Table**). We included for analysis four studies reported by eight records. The flowchart of the selection process is presented **(Fig 1)**.

### Characteristics of included studies

This review included 499 subjects from four RCTs. Two trials included only elderly GBM patients, one included elderly and/or frail GBM patients and one also included patients with a diagnosis of high-grade glioma (HGG). We selected only the elderly GBM subgroup of the last two trials. The characteristics of the population included in this review were remarkably similar. Two RCTs (Roa et al., 2004 and Roa et al., 2015/ Castro et al., 2017) were of non inferiority tests.

The main characteristics of the studies are described in **Table 1.**

**Table 1:** Main characteristics of included RCTs.

Study	Country	Duration	Sample size	Population	Mean/median age of participants (years)	PS/KPS	Ressection (N)	Follow up (months)	Comparators	Funding	Conflict of interest
**Bleehen 1991**	United Kingdom South Africa	1983–1988	443 Included in this review: 140	AA + GBM Included in this review: GBM≥60y	subgroup: GBM≥60y (60–73) mean: NR	WHO 0–4 (all patients) GBM≥60y = NR	Biopsy = 192 Partial = 179 Total = 72 (all patients) GBM≥60y = NR	36	arm 1 = 45Gy/20fr arm 2 = 60Gy/30fr	Medical Research Council Brain Tumour Working Party	
**Roa 2004**	Canada	1996–2001	100	GBM≥60y	Arm 1 mean = 72.4y SD = 5.4 arm 2 mean = 71.0y SD = 5.5	KPS 50–100 median = 70	Biopsy = 37 Partial = 49 Total = 9	24	arm 1 = 60Gy/30fr arm 2 = 40Gy/15fr	Alberta Cancer Board	
**Malmström 2012**	Austria, Denmark, France, Norway, Sweden, Switzerland Turkey	2000–2009	342* 198 included in this review: (subgroup RT x HRT)	GBM≥60y (Oct 15, 2004 -changed to ≥65y)	arm 2 median = 70y (60–83) arm 3: median = 70y (60–80)	PS 0–2 (accepted: if neurological deficits gave performance score of 3)	Biopsy = 53 Partial +Total = 145 (HRT and RT only)	36	arm 1 = TMZ (not included) arm 2 = 34Gy/10fr arm 3 = 60Gy/30fr	Lion’s Cancer Research Foundation, University of Umeå, Swedish Cancer Fonden, Sweden.	Merck. Merck Sharp and Dohme Schering-Plough
**Roa 2015 /**	Belarus, Brazil, Georgia, Greece, India, Ireland, Poland, Thailand, Tunisia	2009–2011	98 61 included in this review: subgroup age ≥ 65y	GBM ≥65y and/or frail Included in this review: subgroup: ≥65y	Mean = 70y	KPS: 50–90 (66% ≤70)	Biopsy = 8 Partial = 35 Total = 14 Not defined = 3 (subgroup: ≥65y)	24	arm 1 = 25Gy/5fr arm 2 = 40Gy/15fr	International Atomic Energy Agency (IAEA) Coordinated Research Activities.	
**Castro 2017**											

AA = anaplastic astrocytoma; fr = fraction; GBM = glioblastoma; Gy = Gray; HRT = hypofractionated radiotherapy; IAEA = International Atomic Energy Agency; KPS = Karnosfsky performance status; MRC = Medical Research Council; NCBTSG = The Nordic Clinical Brain Tumour Study Group; NR = not reported; PS = performance status; SD = standard deviation; SRT = standard radiotherapy; TMZ = temozolomide.

* Malmström study: N = 342; patients evaluated in the review: N = 198; considered only the comparison HRT (N = 98) versus SRT (N = 100).

### Risk of bias of included RCT

Presented in **online S1 Fig**

### Results of included studies

We could only perform a quantitative analysis for the OS outcome. For the other three outcomes (AE, PFS and QoL), (a) the available data were not sufficient or (b) they were measured or presented heterogeneously, not allowing for a quantitative synthesis.

### Qualitative analysis

Four studies were included (Bleehen et al., 1991; Roa et al., 2004; Malmström et al., 2012; Roa et al., 2015 / Castro et al., 2017) [26–30]. Castro et al., 2017 reported a post-hoc subgroup analysis (elderly patients) from the original RCT published in Roa et al., 2015. We used the data from Castro et al. (only elderly patients). Roa’s trial evaluated a more generalized population(they included frail patients aged below 65—not evaluated in our review).

Their outcomes are detailed in **Table 2** and briefly discussed below.

**Table 2:** Outcomes evaluated in the included RCTs.

Author pub (year)	N	Interventions compared	OS (months) CI 95%	HR 95%CI	PFS 95%CI	AE	QoL
**Bleehen, 1991**	49	arm1 = 45Gy/20 fr	missing	1 [0.54, 1.89] (GBM)	NE	For the entire population: Evaluationonly during RT >5% = nausea and vomiting No major difference between arms.	WHO PS: only for the entire population (each visit during follow-up) % patients WHO PS 0–1 = fairly constant: ≥ 51% in 45Gy arm ≥ 45% in 60Gy arm
	91	arm 2 = 60Gy/30fr	missing				
**Roa, 2004**	47	arm 1 = 60Gy/30fr	5.1	0.89 [0.59, 1.36]	NE	NE	Median KPS: HRT versus SRT KPS [IQR] KPS [IQR] Baseline = 70 [60, 80] versus 70 [60, 80] 3 w = 65 [50, 80] versus 70 [60, 80] 6 w = 70 [60, 80] versus 70 [50, 80] 3 m = 70 [50, 70] versus 65 [50, 80] 6 m = 60 [60, 70] versus 60 [40, 70] FACT-Br: N too low to compare groups.
	48	arm 2 = 40Gy/15fr	5.6				
**Malmström, 2012**	x	* (arm 1 = TMZ) Not included	Not included	Not included	NE	HRT vs SRT (PP)** Grade 3 = 8 versus 11 Grade 4 = 5 versus 3 Grade 5 = 0 versus 1	EORTC QoL (mean—change)****Global health status: 3w and 3 m 3 m = 44% completed the questionnaire) arm: 60 Gy 34Gybaseline: 0.00 0.00
	98	arm 2 = 34Gy/10fr	7.5 [6.5, 8.6]	0.85 [0.64, 1.12]			
							6 w -0.67–2.27
							min -7.52–7.98
							max 8.85 3.43
							3m -7.07–4.27
							min -16.26–10.65
							max 2.12 2.11
	100	arm 3 = 60Gy/30fr	6.0 [5.1, 6.8]	1.0			
**Roa,2015/ Castro, 2017 (Data from Castro, 2017)**	26	arm 1 = 25Gy/5fr	6.8 [4.5, 9.1]	1.10 [0.66, 1.83] (Roa/ Castro)***	4.3 [2.6, 5.9]	Acute toxicity (subgroup: ≥65y) Grade ≥ 3 = 0	EORTC QoL (Mean (±SD), score) **** (subgroup: ≥65y) Global health status: baseline: N = 23 versus 33 47.1 (±22.5) versus 50.3 (±17.2), p = 0.56 4 w: N = 20 versus 20 51.7 (±18.0) versus 48.3 (±19.8), p = 0.58 8 w = N = 12 versus 12 48.6 (±18.4) versus 48.6 (±15.4), p > 0.99
	35	arm 2 = 40Gy/15fr	6.2 [4.7, 7.7]		3.2 [0.1, 6.3]		

EORTC QoL) = European Organization for Research and Treatment of Cancer Quality of Life Questionnaire 30 (EORTC QLQ-30), with the brain cancer module 20 (QLQ-BN20); FACT-Br = Functional Assessment of Cancer Therapy—Brain; fr = fraction; GBM = glioblastoma; Gy = Gray; HRT = hypofractionated radiotherapy; KPS = Karnosfsky Performance Status; NE = not evaluated in the trial; PP = per protocol; PS = performance status (WHO); RT = radiotherapy; SD = standard deviation; SRT = standard radiotherapy; w = week; y = year.

* TMZ = arm 1(not evaluated in this review);)

** AE—only for the comparison HRT versus RT (TMZ excluded)

*** Castro—personal communication

****; QoL: Categorical scales are transformed to linear scales from 0 to 100.

#### Overall survival (OS)

All four RCTs evaluated this outcome. One RCT compared two schemes of HRTs (Roa et al., 2015/Castro et al., 2017) and the other three, HRT versus SRT (Bleehen et al., 1991; Roa et al., 2004; Malmström et al., 2012). The three RCTs that compared HRT versus SRT showed no evidence of a difference in survival, except in a subset analysis of the group aged > 70 years, for which hypofractionation was shown to be superior (Malmström et al., 2012). The authors suggest the completion of treatment as a possible reason for this superiority. The elderly population of Bleehen et al. (1991) was part of a subgroup of malignant gliomas (astrocytoma grade 3 and GBM). The only study comparing two HRTs (Roa et al., 2015/Castro et al., 2017) also found no strong evidence of a difference between arms.

#### Adverse events (AE)

In this review, we only evaluated AE ≥ grade 3. Two RCTs evaluated this outcome at follow-up (Malmström et al., 2012 and Roa et al., 2015/Castro et al., 2017) and one (Bleehen et al., 1991) only during RT and not specifically for the group of elderly patients with GBM. Roa et al., 2004 did not evaluate AE. For the general population of Bleehen et al., during RT, nausea and vomiting (no report of grade) was the only AE observed at a frequency of 5% or higher. For this outcome, there was no evidence of a difference in the two interventions. Malmström et al. 2012 reported, in the HRT and SRT arms, respectively, AE grade 3 in 8/95 versus 11/95 patients; AE grade 4 in 5/95 versus 3/95 patients; and AE grade 5 in 0/95 versus 1/95 patients (analysis per protocol). In the SRT arm, infection (grade ≥3 in 7/95 patients), with one fatal case, and seizures (grade ≥3 in 8/95 patients) were predominant. In the HRT arm, there were more thromboembolic events (grade ≥3 in 6/95 patients). Roa et al., 2015 / Castro et al., 2017 did not report AE ≥ grade 3.

#### Progression-free survival (PFS)

One RCT evaluated and reported this outcome (Roa et al., 2015/Castro et al., 2017). The median PFS was similar between the two arms.

#### Quality of life (QoL)

Four RCTs evaluated this outcome. The authors used different methods and time points to evaluate QoL. Only two of them applied the European Organization for Research and Treatment of Cancer Quality of Life Questionnaire Core 30 (EORTC QLQ-30), and brain cancer-specific (QLQ-BN-20) questionnaire (Malmström et al., 2012 and Roa et al., 2015/Castro et al., 2017).

Bleehen et al. did not report this outcome, selectively, for the elderly GBM patients. For the general population of the study, they used the WHO PS scale, which stayed fairly constant during follow-up.

Roa et al. (2004) measured KPS at baseline, during RT (3 and 6 weeks), and every three months after that. There was no evidence of a difference between the arms. Also, KPS remained fairly constant during follow-up, in both arms.

Malmström et al. 2012 measured the change of values in six weeks and three months. The results (A. Malmström, unpublished data) showed a reduction after three months of 4.3 (-10.7; 2.1) points in the 34 Gy arms vs. 7.1(-16.3; 2.1) points in the 60 Gy arm.

Roa et al., 2015 / Castro et al., 2017 evaluated all patients at the baseline and four and eight weeks post-treatment. The QoL score decreased in both intervention groups, but there was no strong evidence of a difference between them. They planned to assess QoL after three months, but the questionnaires were answered only by 44% of patients, not enough for conclusions.

### Pairwise meta-analysis

We identified four RCTs, with four different pairwise comparators. Thus, we did not perform any pairwise meta-analysis.

### Network meta-analysis (NMA)

#### Geometry

**Fig 2:**
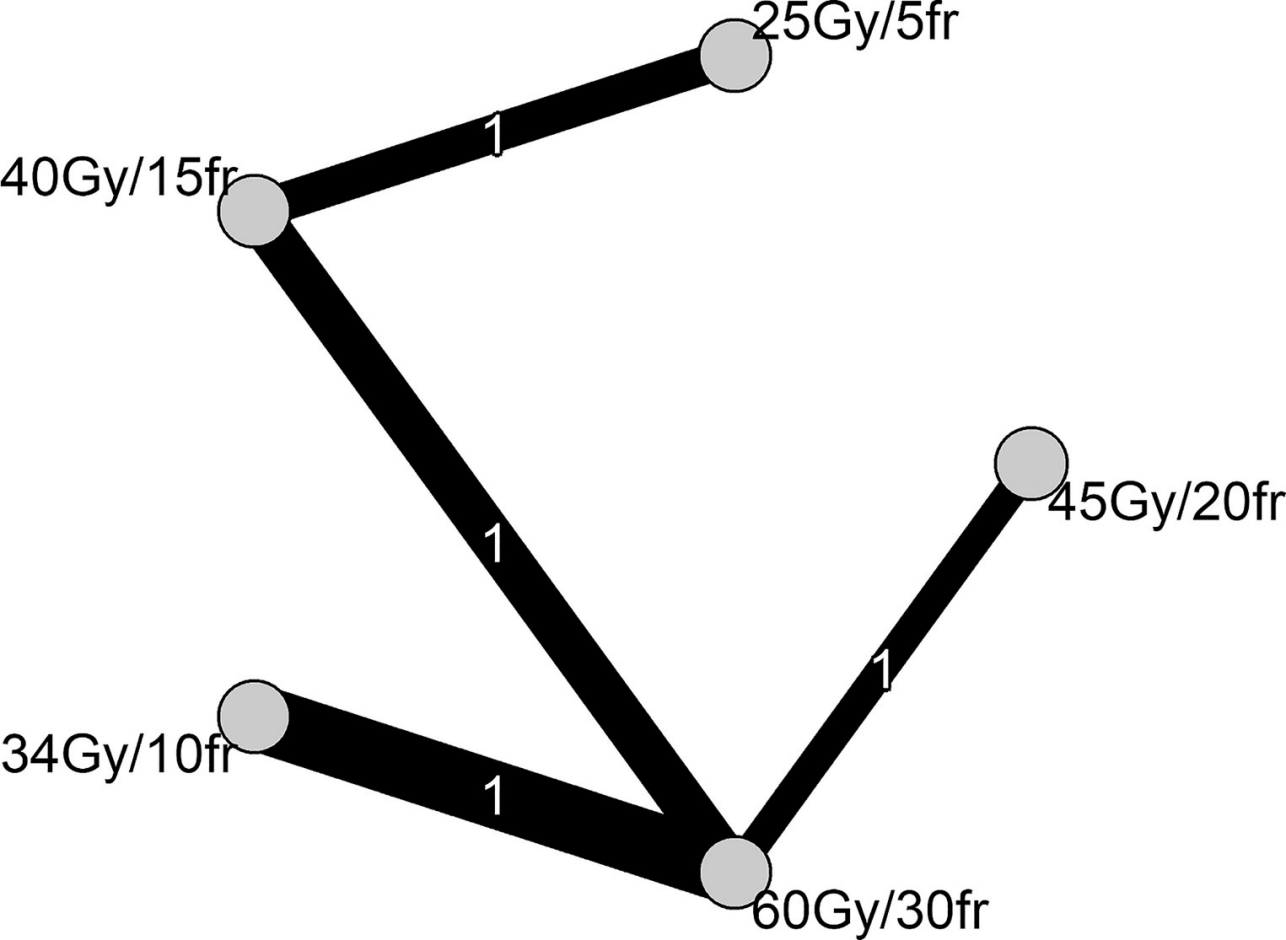
Network diagram.

The network diagram (Fig 2) represents the comparisons between different monotherapy HRT schemes. The thickness of the lines is proportional to the standard error of the estimated effect size for each comparison.

We assessed the transitivity assumption by comparing the studies in terms of important effect modifiers. We did not find material differences across the studies.

We did not assess heterogeneity or inconsistency due to having only one study per comparison, and no loop in the network [22].

**Fig 3:**
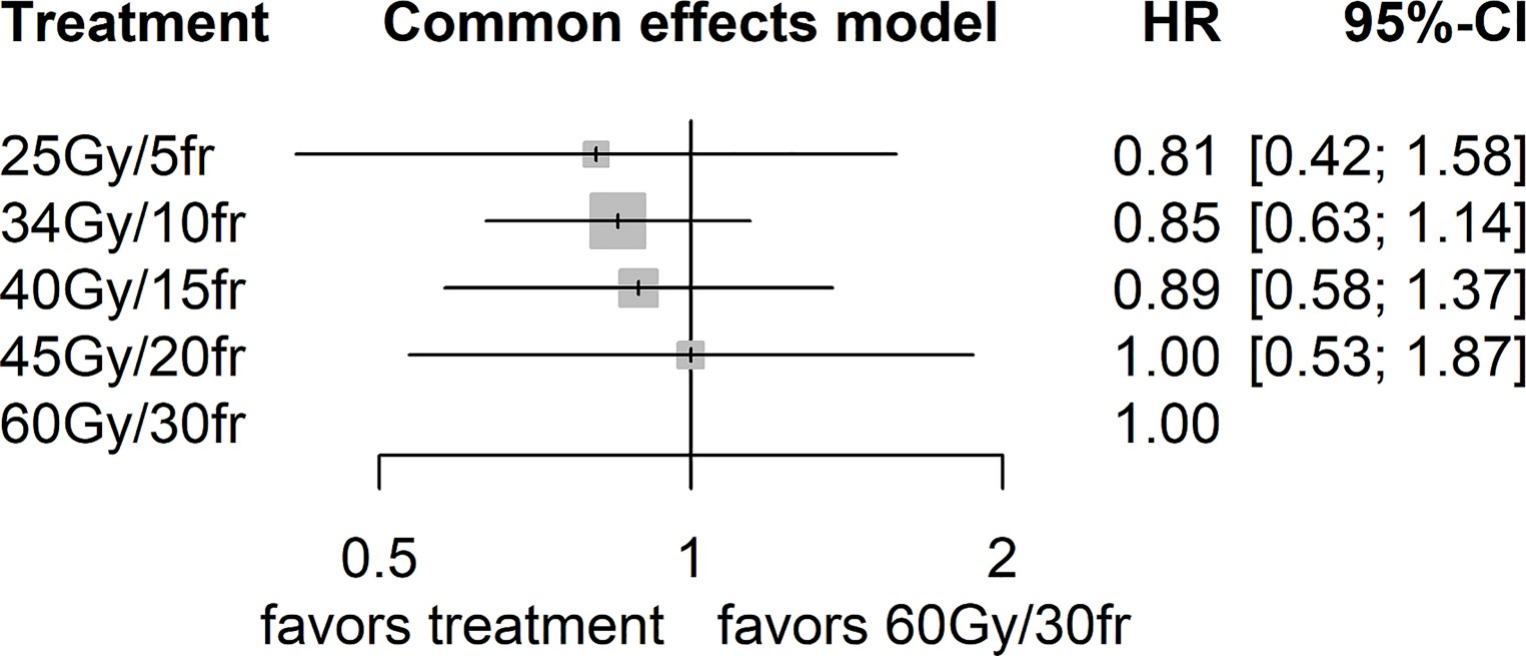
Results from NMA for OS.

The comparisons of different HRTs versus SRT (60 Gy/30fr) for OS are presented in Fig 3.

The HRs of the indirect comparisons were: 40 Gy versus 45 Gy (HR: 0.89; 95%CI 0.42, 1.91); 34 Gy versus 45 Gy (HR: 0.85; 95%CI 0.43, 1.70); 25 Gy versus 45 Gy (HR: 0.81; 95%CI 0.32, 2.02); 34 Gy versus 40 Gy (HR: 0.95; 95%CI 0.57, 1.61); 25 Gy versus 34 Gy (HR: 0.95; 95%CI 0.46, 1.97). Overall, we did not find any strong evidence of difference between the HRTs evaluated (**Table 3**).

**Table 3:** League table of the NMA results for the outcome OS.

**25 Gy/5 fr**	.	0.91 [0.55, 1.52]	.	.
0.95 [0.46, 1.98]	**34 Gy/10 fr**	.	.	0.85 [0.64, 1.12]
0.91 [0.55, 1.51]	0.95 [0.57, 1.61]	**40 Gy/15 fr**	.	0.89 [0.59, 1.36]
0.81 [0.32, 2.03]	0.85 [0.43, 1.70]	0.89 [0.42, 1.91]	**45 Gy/20 fr**	1.00 [0.55, 1.52]
0.81 [0.41, 1.58]	0.85 [0.63, 1.14]	0.89 [0.58, 1.37]	1.00 [0.53, 1.87]	**60 Gy/30 fr**

Comparisons should be read from left to right. The top right cells (dark gray) give the direct estimates. An HR below 1.0 in the top right cells favours the hypofractionated radiotherapy. The botton left cells (light gray) give the indirect estimates. There was no evidence of a difference between any of the included treatments. *No direct comparison.

The interventions were ranked according to p-scores as follows: 25 Gy/5fr (0.65), 34 Gy/10fr (0.64), 40 Gy/15fr (0.53), 45 Gy/20fr (0.38) and 60 Gy/30fr (0.30), with a possibility of change in the positions of treatments (no significant difference). The treatment 25 Gy/5fr is on mean 65% better than other treatments. P-score measures the mean extent of certainty that a treatment is better than other treatments, considering accuracy (22).

#### Subgroup and sensitivity analysis

Subgroup analyses regarding the MGMT marker and the risk of bias were not possible due to the small number of studies evaluated.

#### Certainty of evidence (CINeMA)

Confidence in the results of the network meta-analysis for OS.

Within-study bias (RoB table) of direct comparisons for the OS (objective outcome) was low for all comparisons. As regards indirectness, there were some concerns for the comparison of 45 Gy versus 60 Gy. There were no concerns for the rest. Although Castro et al. was a *post-hoc* analysis, they included two-thirds of the population estimated in the original trial (Roa et al., 2015) and the characteristics of the population and the OS in the subgroup analysis were similar to those of the original study. In the evaluation of imprecision, all results had a large confidence interval due to the small number of patients in each arm. Thus, there were major concerns for all comparisons. We did not observe any significant clinical or methodological heterogeneity between comparators in the NMA. Due to having only one study per comparison, estimating heterogeneity was impossible. Thus, we assumed no concerns for all comparisons. There was no evidence of a lack of transitivity. Incoherence was not assessed.

## Discussion

The elderly population in the four studies was homogeneous for prognosis related to age (mean/median age of 70–72 years) and KPS (≅70) (clinical transitivity between comparisons in the NMA). Elderly GBM patients have a homogenerous response to the same treatment, among different age ranges [7].

The total number of patients evaluated in this review was 499 (Bleehen et al., 1991, n = 140; Roa et al.,2004, n = 100; Malström et al.,2012, n = 198; Roa et al.,2015/Castro et al., 2017, n = 61). All four studies were RCTs (according to our inclusion criteria). Bleehen et al.,1991 and Roa et al.,2004 might be biased as they are old studies and more recently new methodological rules were established to decrease possible bias [13]. Also, all RCTs used an old classification of gliomas. In the 2016 WHO classification, the presence or absence of the molecular marker isocitrate dehydrogenase mutation (IDHmut) defines two different types of GBM (secondary and primary, respectively), with different prognosis, not used in the old classification. This mark is almost nonexistent in the elderly GBM patients, and does not change the results in this population. We evaluated only elderly patients with a proven histology of GBM, and most likely, in this age range, the same diagnosis would be maintained (primary GBM) in the new classification. We asked for data from the patients refered as aged over 60 (N = 140) in Bleehen et al.,1991 due to there being no specific data for the elderly GBM patients in their publication. The contact from the MRC study sent a form to allow us to receive the data, which was completed and returned, but we did not receive any answer after that. Two results were from subgroups of RCTs (Bleehen et al., 1991 and Castro et al., 2017), with a small number of patients in each arm. Although Castro et al. (2017) was a subgroup analysis, approximately two-thirds of the patients from the original study participated in the subgroup analysis, and the results observed were similar to those found in the original study (7.9 months, 95% CI 6.3, 9.6 versus 6.4 months, CI 95% 5.1, 7.6 in arms 1 and 2, respectively; p = 0.988).

In Roa et al.,2004, the number of patients included was inferior to the pre-calculated number for a power of 80% (N calculated was 202). Malström et al. was terminated after including 342 patients, lower than the number calculated for a 90% power (160 patients per arm).

### Concordances and disagreements with other studies or reviews

The results of retrospective studies are not substantially different from those observed in this systematic review (which included only randomized studies). Harris et al. retrospectively assessed 108 GBM patients aged 75 or older treated with IMRT (HRT or SRT with doses of 40Gy or 60Gy) and observed a median OS of 6.3 months and no impact of the RT dose used [31].

Some observational studies reported larger OS with the use of SRT versus HRT. However, the second treatment was predominantly given to elderly and with worse prognosis patients, with consequent bias [9]. When Bingham et al. excluded patients who died within the first 90 days, to reduce bias related to the choice of HRT (40Gy) in patients with poor prognosis, they observed similar efficacy between both treatments in elderly patients with GBM [10].

In regard to the recent randomized study (NCIC/EORTC) evaluating HRT (40Gy) alone or in association with TMZ, the HRT alone group had a median OS of 7.6 months, not very different from the results in this review [13].

A Cochrane systematic review published in 2016 presented a subgroup analysis comparing HRT and SRT in elderly patients. In that, they considered treatments included in our review (Roa et al., 2004 and Malmström et al., 2012). They found the treatments to be equally effective except for patients aged 70 or older (discussed in our review). These patients had lower OS when treated with SRT. The authors considered the certainty related to the evidence for this subgroup to be high [32].

In all the studies, there was a good tolerance to treatment.

#### Strengths

To the best of our knowledge, this is the first review focused on an indirect comparison of different monotherapy HRT schemes in the treatment of elderly patients with newly diagnosed GBM, in contrast to other published reviews on the subject of treatment in the elderly. This review followed the recommendations of the Cochrane Handbook for Systematic Reviews of Interventions, at all stages. There were no date or language limitations on the search strategy in an attempt to minimize the risk of bias due to study omission. All authors of the included studies were contacted to provide additional information. Dr. Malmström provided us with QoL data.

All included studies were RCTs and were high GRADE for the only outcome for which we did a quantitative analysis (OS), as the impact of non-blinding studies is not relevant for objectives outcomes. In addition, even though we did not identify an HRT scheme that was more effective in terms of OS, we provided a ranking of HRT schemes in an attempt to help identify the most plausible comparisons for future studies.

#### Limitations

Despite all its methodological rigor, this review presents limitations related to the small number of RCTs identified, resulting in only one study contributing to each comparison, with an insufficient number of patients for allowing precise estimates. As each direct comparison arm had only one study, it is likely that any bias in these studies influenced the results of the network meta-analysis [33]. In Bleheen et al.,1991, there were no specific data for the elderly patients, leading to a limited analysis of the outcomes. We did not consider the old classification method for the histopathological diagnosis of GBM a limiting factor, since, in this age group, the likelihood of alteration in the diagnosis would be improbable.

Another limitation was the infeasibility of a network meta-analysis of the other proposed outcomes (AE, QoL and PFS). This was because they were heterogeneously reported or there was a large amount of missing data in the long-term assessment.

These restraints support the need for additional RCTs comparing different HRTs to get a consensus on the best regimen for this population.

## Conclusion

This systematic review evaluated a different hypofractionated regimen for the treatment of elderly GBM patients. We included a total of 499 participants in four RCTs, three comparing HRT to SRT and one comparing two HRT regimens. Due to the scarcity of available data and the heterogeneity of how the outcomes were measured and presented, quantitative synthesis was possible only for OS. The network meta-analysis summarized the comparative effects of four different hypofractionated radiotherapy regimens and did not find any evidence of a difference between them. Due to the presence of only one study per comparison and the small number of patients evaluated, the review did not have enough power to detect possible differences among the various hypofractionated radiotherapy schemes.

In the qualitative evaluation concerning AE and QoL (evaluated by different methods and times), HRT and SRT did not result in significant worsening these outcomes after treatment.

Our analysis was based on a small number of studies. For a definitive conclusion and as an implication for futher research, there is a need for more well-planned and well-conducted RCTs comparing different fractions of radiotherapy in the elderly population to determine the best regimen in terms of efficacy, safety and quality of life. Also, due to the poor prognosis of GBM, mostly in elderly patients, their inclusion in a clinical trial will enable the current knowledge regarding the efficacy of new treatments for this neglected population to be expanded.

A better knowledge of the different molecular characteristics of the tumor and standardization of the definition of the elderly population will help in evaluating the differences in the responses associated with these characteristics, identifying who will benefit from a specific regimen.

## Supporting information

S1 Checklist (DOCX)

S1 Table Search strategy. (DOCX)

S2 Table Excluded studies with reasons. (TIF)

S1 Fig Risk of bias graph. (TIF)
