# Supplementary material for: Hypofractionated radiotherapy for newly diagnosed elderly glioblastoma patients: A systematic review and network meta-analysis
Source: PLoS One. 2021 Nov 4;16(11):e0257384. doi: 10.1371/journal.pone.0257384 (PMC8568110; doi:10.1371/journal.pone.0257384)
Supplement: S1 Checklist — (DOCX) [file pone.0257384.s001.docx]

**PRISMA NMA Checklist of Items to Include When Reporting A Systematic Review Involving a Network Meta-analysis**

| **Section/Topic** | **Item #** | **Checklist Item** | **Reported on Page #** |
| --- | --- | --- | --- |
| **TITLE** |  |  |  |
| Title | 1 | **Hypofractionated radiotherapy for newly diagnosed elderly glioblastoma patients - a systematic review and network meta-analysis** | ***Tittle pg 1*** |
|  |  |  |  |
| **ABSTRACT** |  |  |  |
| Structured summary | 2 | **Objective**: To evaluate different hypofractionated radiotherapy (HRT) regimens for newly diagnosed elderly glioblastoma (GBM) patients. **Methods:** We performed a systematic review with network meta-analysis (NMA), including search on CENTRAL, Medline, EMBASE, CINAHL, clinical trial databases and manual search. Only randomized clinical trials (RCTs) were included. Primary outcomes: overall survival (OS) and adverse events (AE). Secondary outcomes: progression-free survival (PFS) and quality of life (QoL). We used Cochrane risk of bias (RoB) table for assessing individual studies and CINeMA for evaluating the certainty of the final body of the evidence. **Results:** Four RCTs (499 patients) were included. For OS, the estimates from NMA did not provide strong evidence of a difference between the HRTs: 40 Gray(Gy) versus 45 Gy (HR: 0.89; CI 95%: 0.42, 1.91); 34 Gy versus 45 Gy (HR: 0.85; CI 95% 0.43, 1.70); 25 Gy versus 45 Gy (HR: 0.81; CI 95% 0.32, 2.02); 34 Gy versus 40 Gy (HR: 0.95; CI 95% 0.57, 1.61); 25 Gy versus 34 Gy (HR: 0.95; CI 95% 0.46, 1.97). We performed qualitative synthesis for AE and QoL, due to data data scarcity and clinical heterogeneity among studies. The four studies reported similar QoL (assessed by different methods) between arms. One RCT reported grade ≥ 3 AE, with no evidence of a difference between arms. PFS was reported in one study (25 Gy versus 40 Gy), with no evidence of a difference between arms**. Conclusion**: This review found no evidence of a difference between the evaluated HRTs for efficacy and safety.  **Other:**  PROSPERO database and available from <http://www.crd.york.ac.uk/PROSPERO/display_record.php?ID=CRD42018100600>  Funding: OE is supported by the Swiss National Science foundation (SNF), grant 180083 | Pg 2  Pgs 4,19 |
|  |  |  |  |
| **INTRODUCTION** |  |  |  |
| Rationale | 3 | Glioblastoma (GBM) is the most common primary malignant brain tumor in adults (47.7%) and accounts for 56.6% of all gliomas  Elderly patients usually suffer from additional co-morbidities and are associated with worse prognosis ^6^. For this reason, clinical trials in patients with GBM traditionally excluded elderly patients. Population studies showed that elderly patients usually have a less intense treatments. However, when this differential choice of treatment was accounted for in the analyses, the difference between the outcomes in the different treatments disappeared  The NCIC/EORTC phase III trial assessed patients ≥ 65 years old and  [Eastern Cooperative Oncology Group](https://en.wikipedia.org/wiki/Eastern_Cooperative_Oncology_Group) (ECOG) performance status 0-2 and randomized them to receive concomitant TMZ (75 mg/m2/day) and HRT (40 Gy in 15 fractions), followed by adjuvant temozolomide (150–200 mg/m2 5 days / 28-day cycle / 12 cycles or until progression) with HRT alone (same schedule.  This treatment is now considered to be the standard of care for good prognosis elderly patients.  However, due to the lack of direct comparison between the HRT schemes, it is not yet possible to define the treatment of choice for this population*.*  *Intention:* Reducing the time of treatment without compromising safety and efficacy | ***Pgs 3-4*** |
| Objectives | 4 | This study aimed to compare the efficacy and safety of different HRT schemes for elderly patients with newly diagnosed GBM | Pg 4 |
|  |  |  |  |
| **METHODS** |  |  |  |
| Protocol and registration | 5 | PROSPERO database and available from <http://www.crd.york.ac.uk/PROSPERO/display_record.php?ID=CRD42018100600>  . | Pg 4 |
| Eligibility criteria | 6 | **Type of participants (P)**  Subjects with newly diagnosed histologically confirmed GBM (WHO G IV), aged 60 years or older.  **Type of interventions (I+C)**  Pos-operative focal Hypofractionations regimens  **Outcome (O)**  The primary outcomes were: (a) overall survival (OS) defined as the time from diagnosis or randomization to the date of death or last follow-up, and (b) adverse events (Aes) as defined by the World Health Organization (WHO) or the National Cancer Institute Common Terminology Criteria (NCI-CTC). Secondary outcomes were (c) progression-free survival (PFS) defined as the time from diagnosis or randomization to the date of progression (Response assessment in neuro-oncology criteria – RANO) or death and (d) health-related quality of life (QoL), assessed by any validated tool.  **Type of studies (S)**  Randomized clinical trials (RCTs).  **Timing of outcome assessment**  We assessed all stated above outcomes at any time point. However, we only pooled short term (up to three months, inclusive) or long term (more than three months) outcomes. When a study reported an outcome more than once in the same period, we considered the last measurement.  We did not impose restrictions for date, language or status of publication (abstract or full-text). | ***Pgs 4-5*** |
| Information sources | 7 | **Search strategy**  We conducted (January 28, 2019) highly sensitive searches strategies for the following electronic databases: Excerpta Medica dataBASE (Embase, via Elsevier), Cochrane Central Register of Controlled Trials (CENTRAL, via Wiley) and Medical Literature Analysis and Retrieval System Online (MEDLINE, via PubMed). The full search strategies for all databases are available at online Supplementary Table I. We carried additional searches in grey literature ([www.opengrey.eu)](http://www.opengrey.eu)) and clinical trials register databases (<https://www.clinicaltrials.gov> and <https://www.who.int/ictrp/search/en/)>. We conducted manual searches along the reference lists of included studies, review articles and proceedings of the meetings of the American Society of Radiation Oncology (ASTRO) and European Society for Radiotherapy and Oncology (ESTRO). We did not impose restrictions for date, language or status of publication (abstract or full-text).  **Dealing with missing data**  We tried to contact authors or study sponsors if missing data related to the outcomes. | Pgs 5-6 |
| Search | 8 | Medline via Pubmed  #1 "Glioblastoma"[Mesh]  #2 Glioblastoma OR Glioblastomas OR (Astrocytoma, Grade IV) OR (Astrocytomas, Grade IV) OR (Grade IV Astrocytoma) OR (Grade IV Astrocytomas) OR (Glioblastoma Multiforme) OR (Giant Cell Glioblastoma) OR (Giant Cell Glioblastomas) OR (Glioblastoma, Giant Cell) OR (Glioblastomas, Giant Cell)  #3 #1 OR #2  #4 "Dose Hypofractionation"[Mesh]  #5 (Dose Hypofractionation) OR (Hypofractionation, Dose) OR (Radiotherapy Minibeams) OR (Minibeam, Radiotherapy) OR (Minibeams, Radiotherapy) OR (Radiotherapy Minibeam) OR (Hypofractionated Dose) OR (Dose, Hypofractionated) OR (Doses, Hypofractionated) OR (Hypofractionated Doses)  #6 (short* course radiotherapy) OR (short* course RT)  #7 #4 OR #5 OR #6  #8 #3 AND #7  159 hits | Suppl material – all strategies |
| Study selection | 9 | **Selecting studies**  We selected the studies through a two-stage process. During the first stage, titles and abstracts of the retrieved references were evaluated for. In the second stage, full texts of potentially eligible studies were scrutinised against the inclusion criteria. Both stages were carried out independently by two reviewers (SMM and GNM), and a third reviewer (RR) decided, in case of divergencies We used the Rayyan Platform for the selection process (<https://rayyan.qcri.org/>) | Pg 5 |
| Data collection process | 10 | **Collecting data**  For the extraction, we used a standard data collection form for intervention review in RCTs only (Cochrane Library). We excluded duplicates and gathered multiple reports of the same study. Two reviewers (SMM and GN) extracted the data from included studies. A third reviewer (RR) solved any disagreement. We obtained data on possible effect modifiers in the population (inclusion and exclusion criteria, different ranges of age, baseline performance status, MGMT, surgical extent) and interventions (HRT protocols), methods (study design, number of study centers and location, duration of study, date of study, withdrawals), outcomes (primary and secondary, planned and reported) sponsorship/funding, and conflicts of interest of authors. | Pgs 5-6 |
| Data items | 11 | **Main Characteristics evaluated:** Country, study duration, sample size, Population, Mean/median age of participants (years) PS/KPS, Ressection, Follow up (months), Comparators, Funding, Conflict of interest  HRT schemes | pgs 6-7 |
| **Geometry of the network** | **S1** | Geometry:  **Network diagram**  One of the underlying assumptions of NMA is transitivity 18,19. We assessed this assumption by comparing the distribution of the potential effect modifiers across the different pairwise comparisons, i.e. if the treatments characteristics, participants and clinical questions were deemed to be similar across treatment comparisons  In case there were more than one study per comparison, we aimed to synthesise data using a random-effects meta-analysis model for each pairwise treatment comparison and to report the estimated heterogeneity. We performed a frequentist NMA, using the netmeta package in R 20. We used a single parameter to model heterogeneity in the network 21, a common assumption in NMA. | pg 6 |
| Risk of bias within individual studies | 12 | **Assessing the risk of bias of includes studies**  RoB table – individual studies  Two reviewers (SMM, GNM) independently evaluated the risk of bias for each study using the Cochrane Cochrane Risk of Bias (RoB) table 17. An additional reviewer (RR) solved any disagreement.  We judged each outcome separately for the domains blinding of participants and personnel and blinding of outcome assessors and incomplete outcome data.  Used in data synthesis*: Certainty of evidence (****CINeMA***  We used the free online tool CINeMA (Confidence in Network Meta-Analysis Software Institute of Social and Preventive Medicine, University of Bern, 2017- [cinema.ispm.unibe.ch](https://cinema.ispm.unibe.ch/)) 25 to evaluate the certainty of evidence for each pre-specified outcome (<https://journals.plos.org/plosmedicine/article?id=10.1371/journal.pmed.1003082>). Two authors applied the tool and divergencies were solved by a third author. | Pgs 6-7 |
| Summary measures | 13 | For time-to-event data, we used hazard ratios (HRs).  For the toxicity analysis we aimed to use risk ratios (RRs).  For the QOL analysis we aimed to use mean score difference.  We reported results in terms of ‘league-tables’ and generalized forest-plots, where we show the relative effects of each treatment versus the network reference (which we chose to be 60 Gy).  The interventions were ranked according to p-scores  **Ranking:** p-score not informative due to no significant difference between schemes (possibility of change in the positions) | Pg 7 |
| Planned methods of analysis | 14 | We assessed the transitivity assumption by comparing the studies in terms of important effect modifiers. We assessed the extend of statistical heterogeneity in our meta-analyses via the estimated heterogeneity variance parameter (). To assess the inconsistency in the network locally, we aimed to use a back-calculation method 23. To assess inconsistency globally, we used the design-by-treatment inconsistency model. This model accounts for design inconsistency (e.g., when two-arm and three-arm trials give different results) as well as loop inconsistency (i.e., the disagreement between direct and indirect evidence).  In case we found comparisons with ten or more studies per comparison, we aimed to produce a contour-enhanced funnel plot to explore whether results in imprecise trials differ systematically from results in more precise trials. We aimed to use an Egger’s test to test for funnel-plot asymmetry, aiming to assess the possibility of small-study effects and publication bias | Pg 7 |
| **Assessment of Inconsistency** | S2 | To assess the inconsistency in the network locally, we aimed to use a back-calculation method 23. To assess inconsistency globally, we used the design-by-treatment inconsistency model. This model accounts for design inconsistency (e.g., when two-arm and three-arm trials give different results) as well as loop inconsistency (i.e., the disagreement between direct and indirect evidence). | Pg 7 |
| Risk of bias across studies | 15 | Seletive reporting  In case we found comparisons with ten or more studies per comparison, we aimed to produce a contour-enhanced funnel plot to explore whether results in imprecise trials differ systematically from results in more precise trials. We aimed to use an Egger’s test to test for funnel-plot asymmetry, aimint to assess the possibility of small-study effects and publication bias ^24^. | **Pg 7** |
| Additional analyses | 16 | In case there were enough studies, we planned to perform a subgroup analysis by the MGMT and by the risk of bias . | ***Pg 7*** |
|  |  |  |  |
| **RESULTS†** |  |  |  |
| Study selection | 17 | The initial search retrieved 290 records. After eliminating duplicates, we assessed 259 records by reading title and abstracts, and identified 22 records as elegible for evaluation in full-text. After this stage, 15 records were excluded due to various reasons (online supplementary table II). We included for analysis four studies reported by eight records. The flowchart of the selection process is presented (Fig 1).  This review included 499 subjects from four RCTs. Two trials included only elderly GBM patients, one, elderly and/or frail GBM patients and one included also patients with the diagnosis of high grade glioma (HGG). We selected only the elderly GBM subgroup of the last two trials. The RCTs were of non-inferiority tests.  **Flowchart of the studies selection process**  **Table of Included and excluded studies**  **Table: Characteristics of included studies**  **Table: Outcomes evaluated in the included RCTs** | Pg 7-8  Fig 1 Suppl table2  Tables 1, 2 |
| **Presentation of network structure** | **S3** | Geometry of NMA  Fig 2. Network diagram. The thickness of the lines is proportional to the standard error of the estimated effect size for each comparison | ***Fig 2*** |
| **Summary of network geometry** | **S4** | NMA: only OS  Qualitative analysis: other outcomes (pgs 8-12)  Table 2 ( characteristics of RCTs) and Table 3 ( outcomes)  We did not assess inconsistency due to having only one study per comparison, and no loop in the network  *Certainty of evidence (CINeMA)*  Confidence in the results of the network meta-analysis for OS - CINeMA  Within-study bias (RoB Table) of direct comparisons for the OS (objective outcome) was low for all comparisons. Regarding indirectness, there were some concerns for the comparison of 45 Gy versus 60 Gy. There were no concerns for the rest. Although Castro et al. was a *post-hoc* analysis, they included 2/3 of the population estimated in the original trial (Roa et al., 2015) and the characteristics of the population and the OS in the subgroup analysis were similar to that of the original study. In the evaluation of imprecision, all results had a large confidence interval because of a small number of patients in each arm. Thus, there were major concerns for all comparisons. We did not observe any significant clinical or methodological heterogeneity between comparators in the NMA. Due to having only one study per comparison, estimating heterogeneity was impossible. Thus, we assumed no concerns for all comparisons. There was no evidence of lack of transitivity. Incoherence was not assessed.  . | ***Table 1 and 2***  ***Pgs***  ***8-12***  ***NMA***  ***13- 14*** |
| Study characteristics | 18 | Main Characteristics evaluated: Country, study duration, sample size, Population, Mean/median age of participants (years) PS/KPS, Ressection, Follow up (months) , Comparators, Funding, Conflict ofinterest  Qualitative analysis  The elderly population in the four studies was homogeneous for prognosis related to age (mean / median age of 70 / 72 years) and KPS (≥70) (clinical transitivity between comparisosn). Elderly GBM patients have a homogenerous response to the same treatment, among different age ranges7.  The total number of patients evaluated in this review was 499 (Bleehen et al. (1991), n = 140, Roa et al. (2004) n = 100, Malström et al. (2012) n = 198; Roa et al. (2015) / Castro et al. (2017) n = 61. All four studies were RCTs (according to our inclusion criteria). Bleehen et al. (1991) and Roa et al. (2004), might be biased as they are old studies and more recently, new methodological rules were established to decrease possible bias 13. Also, they used the old classification of gliomas. In the new WHO classification, the presence or absence of the molecular marker isocitrate dehydrogenase mutation (IDHmut) defines two different types of GBM (secondary and primary respectivelly), with different prognosis, not used in the old classification. This mark is almost non-existent in the elderly GBM patients, and does not change the results in this population. We evaluated only elderly patients with proven histology of GBM, and most likely, at this age range, the same diagnosis would be maintained in the new classification (primary GBM). We asked for data from 140 patients over 60 years of age in Bleehen et al., due to no specific data for the elderly GBM patients in their publication. The contact from the MRC study (Bleehen et al. 1991) sent a form to allow us to receive the data, which was completed and returned, but we did not receive any answer after that. Two results were of subgroups of RCTs (Bleehen et al., 1991 and Castro et al., 2017), with a small number of patients in each arm. Although Castro et al. (2017) was a subgroup analysis, approximately 2/3 of the patients of the original study participated in the subgroup analysis, and the results observed were similar to those found in the original study (7.9 months, 95% CI 6.3, 9.6 versus. 6.4 months, CI 95% 5.1, 7.6 in arms 1 and 2 respectively; p = 0.988).  In Roa et al. (2004) the number of patients included was inferior to the pre-calculated number for a power of 80% (N calculated was 202). Malström et al. was terminated after including 342 patients, lower than that the number calculated for a 90% power (160 patients per arm). | Pgs  8-12, 16  ***Table 1 and 2*** |
| Risk of bias within studies | 19 | Random sequence generation  Four RCTs described the methods used to generate the allocation sequence, and they were considered adequate. We thus classified them as having a low risk of bias for this domain.  Allocation concealment  Four RCTs described the methods used to maintain the confidentiality of the allocation, and the methods were considered adequate. We classified them as low risk of bias for this domain.  Blinding of participants and personnel  Blinding was not done.  For OS, it is unlikely that the results were affected by this fact, and we classified as low risk of bias. Only one RCT (Roa et al. 2015 / Castro et al. 2017) reported SLP, and it is likely that the outcome was affected by not masking, and was considered to have a high risk of bias. EA was reported in 3 RCTs (Bleehen et al. 1991; Malmström et al. 2012; Roa at al. 2015 / Castro at al. 2017), and it is likely that the results were affected by not blinding and was considered to have a high risk of bias. For QoL (reported in all studies), this fact likely affected the outcome, and we considered to have a high risk of bias for this domain.  Blinding of outcome assessment  Three RCTs (Bleehen 1991, Malmström 2012, Roa 2015 / Castro 2017) described the methods used and were considered appropriate. In 1 ECR (Roa 2004), this risk of bias was not described. For the outcome QoL, it was deemed to be uncertain, and if masking was not done, this outcome might have been affected by the lack of blinding.  Incomplete outcome data  There was a low loss rate for OS, in the four RCTs. Only one RCT (Roa 2015 / Castro 2017) reported results in PFS, and there was a low loss rate in this outcome. For the EA outcome, 3 RCTs (Malmström 2012, Roa 2015 / Castro 2017) reported this outcome, and there was a low loss rate. For OS, PFS, and AE domains, it was considered as having a low risk of bias. For the QoL outcome, only one RCT (Bleehen 1991) had a low loss rate and was believed to have a low risk of bias for this domain. The other RCTs presented a high loss rate, and we considered as having a high risk of bias for this domain  Seletive reporting  All RCTs described the proposed outcomes, but Bleehen et al. (1991) and Roa et al. (2004), as they are old studies, have not previously published the protocol. They were thus classified as having an uncertain risk of bias for this domain.  Other sources of bias  We did not find any other source of bias in the four RCTs, and we classified as having a low risk of bias for this domain.  . | Suppl fig 1 + descrtion |
| Results of individual studies | 20 | We could only perform a quantitative analysis for the OS outcome.  We did not perform pairwise meta-analyses due to having only one study per comparison  For the other three outcomes we did a **Qualitative Analysis**  (a) the available data were not sufficient or (b) they were measured or presented heterogeneously, not allowing for a quantitative synthesis.  *.* | ***Pgs***  ***8-13***  Fig 3  Table 3 |
| Synthesis of results | 21 | NMA  League tables and forest plots summarized on fig 3 an table 3  Certainty of evidence summarized on: CINeMA  **Ranking:** p-score –not informative due to no significant difference between schemes ( fig not included)  The HRs of the indirect comparisons were: 40 Gy versus 45 Gy (HR: 0.89; 95%CI: [0.42; 1.91]); 34 Gy versus 45 Gy (HR: 0.85; 95%CI [0.43; 1.70]); 25 Gy versus 45 Gy (HR: 0.81; 95%CI: [0.32; 2.02]); 34 Gy versus 40 Gy (HR: 0.95; 95%CI: [0.57; 1.61]); 25 Gy versus 34 Gy (HR: 0.95; 95%CI: [0.46, 1.97]). Overall, we did not find any strong evidence of difference between the HRTs evaluated.  The interventions were ranked according to p-scores as follows: 25Gy/5fr (0.65), 34Gy/10fr (0.64), 40Gy/15fr (0.53), 45Gy/20fr (0.38) and 60Gy/30fr (0.30), with possibility of change in the positions of treatments (no significant difference) | ***Pg 10***  ***Fig 3***  ***Table 3***  ***Pgs 13-14*** |
| **Exploration for inconsistency** | **S5** | We did not assess inconsistency due to having only one study per comparison, and no loop in the network  See discussion - CINeMA | ***Pg 15*** |
| Risk of bias across studies | 22 | Table 2 ( characteristics of RCTs) and Table 3 ( outcomes)  *Certainty of evidence (CINeMA)*  Confidence in the results of the network meta-analysis for OS - CINeMA  Within-study bias (RoB Table) of direct comparisons for the OS (objective outcome) was low for all comparisons. Regarding indirectness, there were some concerns for the comparison of 45 Gy versus 60 Gy. There were no concerns for the rest. Although Castro et al. was a *post-hoc* analysis, they included 2/3 of the population estimated in the original trial (Roa et al., 2015) and the characteristics of the population and the OS in the subgroup analysis were similar to that of the original study. In the evaluation of imprecision, all results had a large confidence interval because of a small number of patients in each arm. Thus, there were major concerns for all comparisons. We did not observe any significant clinical or methodological heterogeneity between comparators in the NMA. Due to having only one study per comparison, estimating heterogeneity was impossible. Thus, we assumed no concerns for all comparisons. There was no evidence of lack of transitivity. Incoherence was not assessed.  . |  |
| Results of additional analyses | 23 | Subgroup analysis by the MGMT and by the risk of bias were not possible due to the small number of studies evaluated | ***Pg 15*** |
|  |  |  |  |
| **DISCUSSION** |  |  |  |
| Summary of evidence | 24 | This systematic review evaluated different hypofractionated regimen for the treatment of elderly GBM patients. We included a total of 499 participants in four RCTs, three comparing HRT versus SRT and one comparing two HRTs regimen. Due to the scarcity of available data and the heterogeneity of how the outcomes were measured and presented, quantitative synthesis was possible only for OS. The network meta-analysis summarized the comparative effects of four different hypofractionated radiotherapy regimens and did not find any evidence of a difference between them. Due to the presence of only one study per comparison and the small number of patients evaluated, the review did not have enough power to detect possible differences between the various hypofractionated radiotherapy regimen.  In the qualitative evaluation, concerning AE and QoL(evaluated by different methods and times), HRT and SRT did not result in worsening in these outcomes after treatment). Our analysis was based on a small number of studies. For a definitive conclusion and as implication for futher research there is a need for more well-planned and well-conducted RCTs comparing different fractions of radiotherapy in the elderly population to determine the best regimen in terms of efficacy, safety, and quality of life. Also, due to the poor prognosis of GBM, mostly in elderly patients, their inclusion in a clinical trial will enable to expand the current knowledge regarding the efficacy of new treatments for this neglected population. | Pgs  16,  18 |
| Limitations | 25 | Despite all methodological rigor, this review presents limitations related to the small number of RCTs identified, resulting in only one study contributing to each comparison, with insufficient number of patients for allowing precise estimates. As each direct comparison arm had only one study, it is likely that any bias in these studies influenced the results of the network meta-analysis^33^. In Bleheen et al.(1991), there was no specific data for the elderly patients, leading to a limited analysis of the outcomes. We did not consider the old classification method for the histopathological diagnosis of GBM a limiting factor, since, at this age group, the probability of alteration in the diagnosis would be improbable  The old studies did not include MGMTmethylation in their investigation, a known prognostic factor for GBM, and this could result in bias, if it was not evenly distributed between arms ^34-36^.  Another limitation was the infeasibility of a network meta-analysis of the other proposed outcomes (AE, QoL and PFS). This was because they were heterogeneously reported or with high amount of missing data in the long-term assessment.  These restraints support the need for additional RCTs comparing different HRTs to get a consensus on the best regimen for this population. | Pg18 |
| Conclusions | 26 | Results of retrospective studies are not substantially different from those observed in this systematic review (which included only randomized studies). Harris et al. retrospectively assessed 108 GBM patients aged 75 years or older treated with IMRT (HRT and SRT), observed a median OS of 6.3 months and no impact of RT dose used ^31^.  Some observational studies reported larger OS with the use of SRT versus HRT. However, the second treatment was predominantly given to elderly and with worse prognosis patients, with consequent bias ^9^. When Bingham et al. excluded patients who died within the first 90 days, to reduce bias related to the choice of HRT in patients with poor prognosis, they observed similar efficacy between both treatments in elderly patients with GBM ^10^.  Regarding the recent randomized study (NCIC / EORTC), evaluating HRT alone or in association with TMZ, the control group (HRT alone) had a median OS of 7.6 months, not much different from the results in this review ^13^.  A Cochrane systematic review published, in 2016 presented a subgroup analysis comparing HRT versus SRT in elderly patients. There, they considered treatments included in our review (Roa et al. (2004) and Malmström et al. (2012). They found the treatments to be equally effective except for patients 70 years or older. These patients had lower OS when treated with SRT. The authors considered the certainty related to the evidence for this subgroup to be high ^32^.  In all the studies, there was a good tolerance to treatment.  . | Pg18 |
|  |  |  |  |
| **FUNDING** |  |  | Pg18 |
| Funding | 27 | OE is supported by the Swiss National Science foundation (SNF), grant 180083  Conflicts of interest/Competing interests: none declared.  . |  |
