# Supplementary figures and images for: Hypofractionated radiotherapy for newly diagnosed elderly glioblastoma patients: A systematic review and network meta-analysis
Source: PLoS One. 2021 Nov 4;16(11):e0257384. doi: 10.1371/journal.pone.0257384 (PMC8568110; doi:10.1371/journal.pone.0257384)

**Suppl Table 1. Search strategy**


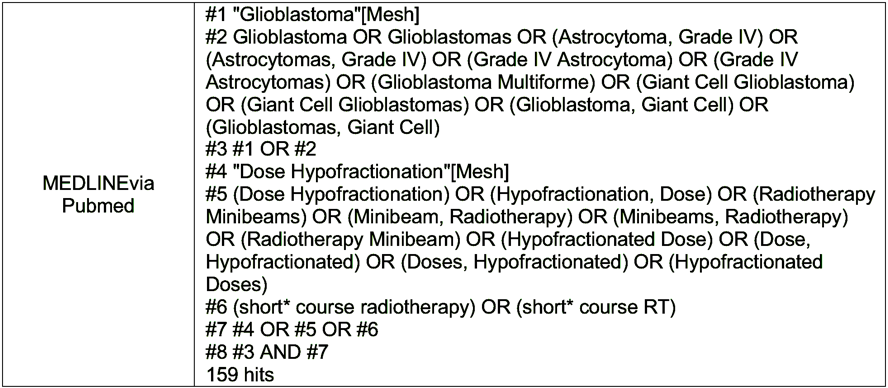


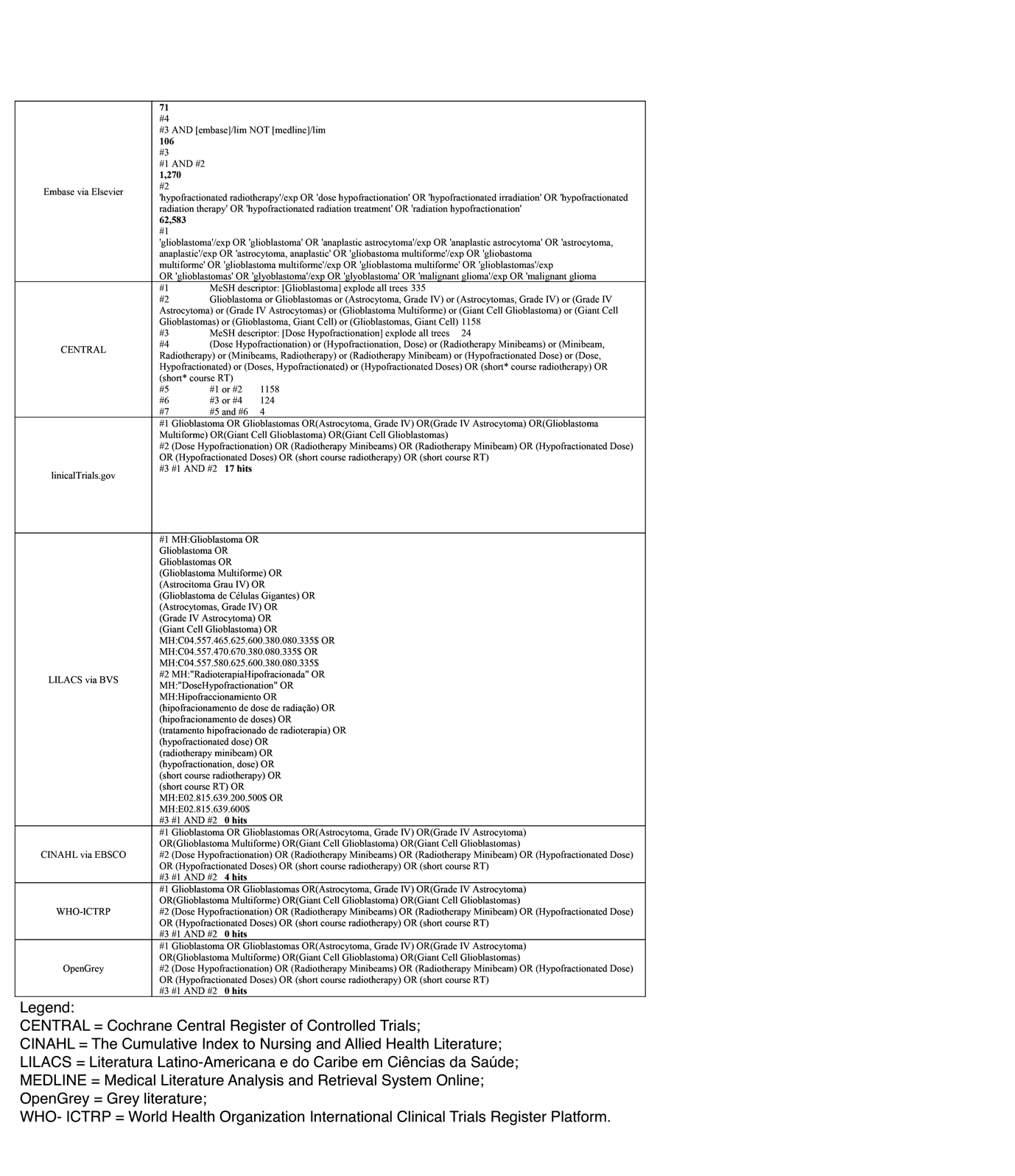

Supplement: S1 Table — (DOCX) [file pone.0257384.s002.docx]

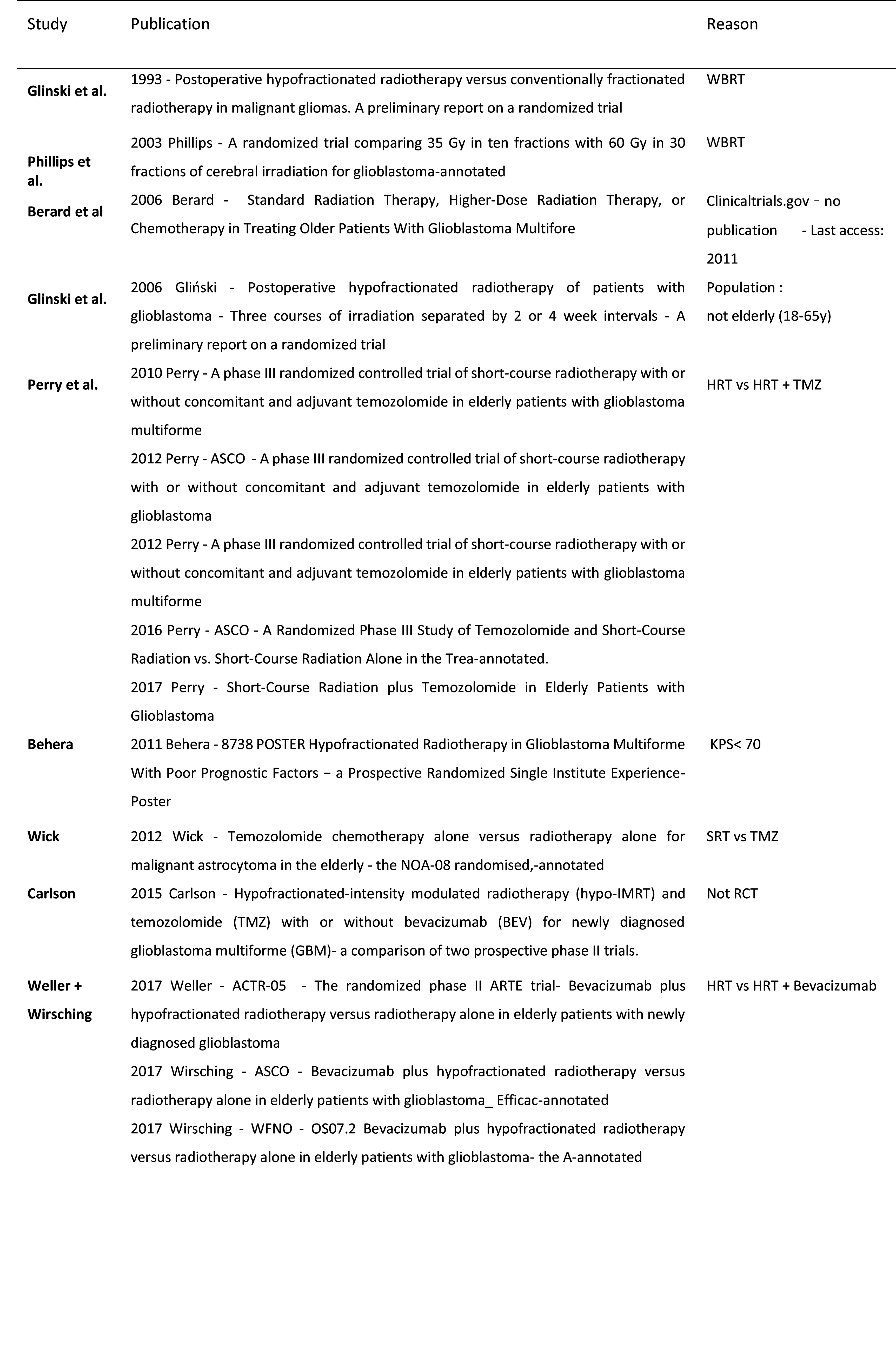

Supplement: S2 Table — (TIF) [file pone.0257384.s003.tif]

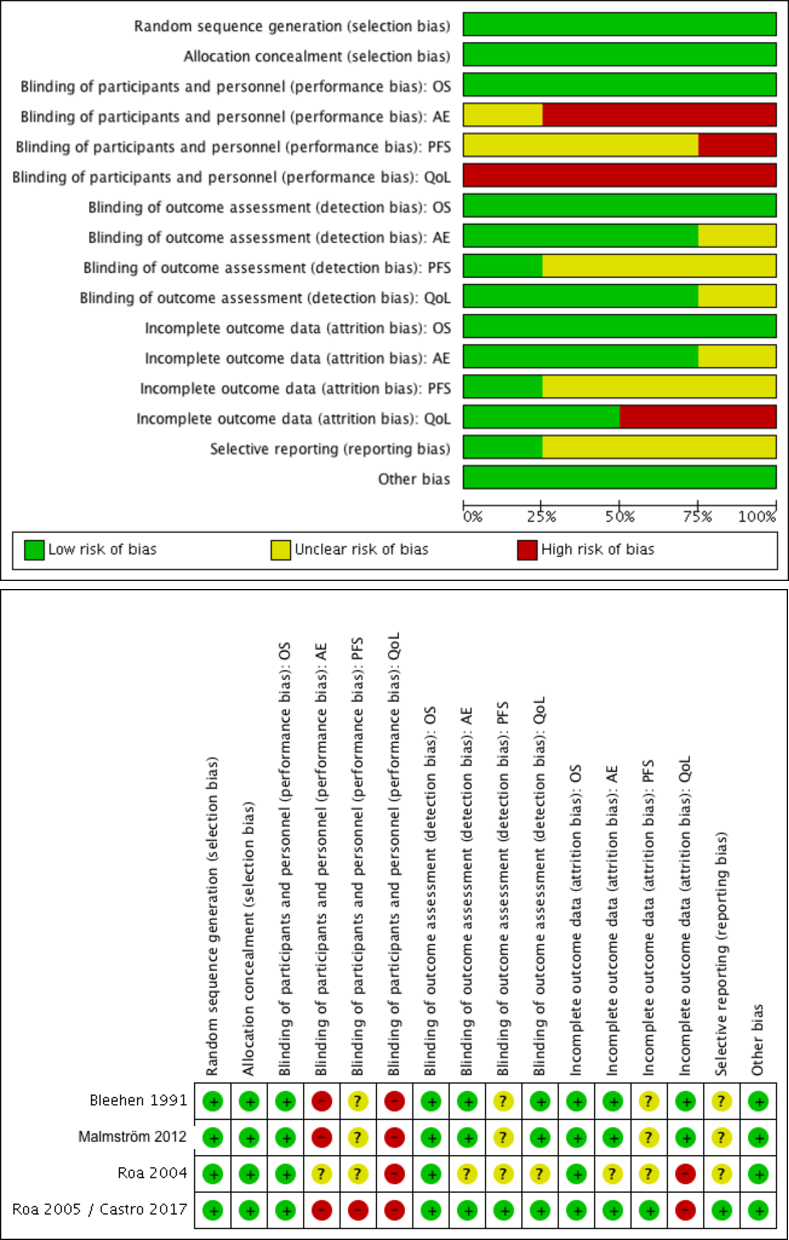

Supplement: S1 Fig — (TIF) [file pone.0257384.s004.tif]
